# Use of whole-genome sequencing to identify clusters of *Shigella flexneri* associated with sexual transmission in men who have sex with men in England: a validation study using linked behavioural data

**DOI:** 10.1099/mgen.0.000311

**Published:** 2019-11-04

**Authors:** Holly D. Mitchell, Amy F. W. Mikhail, Anaïs Painset, Timothy J. Dallman, Claire Jenkins, Nicholas R. Thomson, Nigel Field, Gwenda Hughes

**Affiliations:** ^1^​ Centre for Molecular Epidemiology and Translational Research, Institute for Global Health, University College London, London, UK; ^2^​ National Institute for Health Research Health Protection Research Unit (NIHR HPRU) in Blood Borne and Sexually Transmitted Infections, London, UK; ^3^​ National Infection Service, Public Health England, London, UK; ^4^​ National Institute for Health Research Health Protection Research Unit (NIHR HPRU) in Gastrointestinal Infections, Liverpool, UK; ^5^​ Parasites and Microbes, Wellcome Trust Sanger Institute, Hinxton, UK; ^6^​ Department of Pathogen Molecular Biology, London School of Hygiene and Tropical Medicine, London, UK

**Keywords:** whole-genome sequencing, surveillance, England, *Shigella*

## Abstract

Since the 1970s, shigellosis has been reported as a sexually transmissible infection, and in recent years, genomic data have revealed the breadth of *Shigella* spp. transmission among global networks of men who have sex with men (MSM). In 2015, Public Health England (PHE) introduced routine whole-genome sequencing (WGS) of *Shigella* spp. to identify transmission clusters. However, limited behavioural information for the cases hampers interpretation. We investigated whether WGS can distinguish between clusters representing sexual transmission in MSM and clusters representing community (non-sexual) transmission to inform infection control. WGS data for *Shigella flexneri* from August 2015 to July 2017 were aggregated into single linkage clusters based on SNP typing using a range of SNP distances (the standard for *Shigella* surveillance at PHE is 10 SNPs). Clusters were classified as ‘adult male’, ‘household’, ‘travel-associated’ or ‘community’ using routine demographic data submitted alongside laboratory cultures. From August 2015 to March 2017, PHE contacted those with shigellosis as part of routine public-health follow-up and collected exposure data on a structured questionnaire, which for the first time included questions about sexual identity and behaviour. The questionnaire data were used to determine whether clusters classified as ‘adult male’ represented likely sexual transmission between men, thereby validating the use of the SNP clustering tool for informing appropriate public-health responses. Overall, 1006 *S *. *flexneri* cases were reported, of which 563 clustered with at least one other case (10-SNP threshold). Linked questionnaire data were available for 106 clustered cases, of which 84.0 % belonged to an ‘adult male’ cluster. At the 10-SNP threshold, 95.1 % [95 % confidence interval (CI) 88.0–98.1%] of MSM belonged to an ‘adult male’ cluster, while 73.2 % (95 % CI 49.1–87.5%) of non-MSM belonged to a ‘community’ or ‘travel-associated’ cluster. At the 25-SNP threshold, all MSM (95 % CI 96.0–100%) belonged to an ‘adult male’ cluster and 77.8 % (95 % CI 59.2–89.4%) of non-MSM belonged to a ‘community’ or ‘travel-associated’ cluster. Within one phylogenetic clade of *S. flexneri *, 9 clusters were identified (7 ‘adult male’; 2 ‘community’) using a 10-SNP threshold, while a single ‘adult male’ cluster was identified using a 25-SNP threshold. Genotypic markers of azithromycin resistance were detected in 84.5 % (294/348) of ‘adult male’ cases and 20.9 % (9/43) of cases in other clusters (10-SNP threshold), the latter of which contained gay-identifying men who reported recent same-sex sexual contact. Our study suggests that SNP clustering can be used to identify *Shigella* clusters representing likely sexual transmission in MSM to inform infection control. Defining clusters requires a flexible approach in terms of genetic relatedness to ensure a clear understanding of underlying transmission networks.

## Data Summary

fastq reads from all sequences can be found under Public Health England Pathogens BioProject PRJNA315192 at the National Center for Biotechnology Information Read Archive, available at https://www.ncbi.nlm.nih.gov/bioproject/315192.

**Impact Statement**

In recent years, genomic data have been used to describe the global spread of antimicrobial-resistant *Shigella* among large networks of men who have sex with men (MSM). To facilitate the detection of transmission clusters, Public Health England implemented whole-genome sequencing (WGS) for national surveillance of *Shigella *. SNP typing is used to facilitate cluster detection and ensure timely public-health action. However, interpretation is hindered by the lack of behavioural data. In this study, we use robust data on sexual identity and behaviour linked to individuals presenting with *Shigella flexneri* infections to validate a real-time SNP clustering algorithm. This is the first time that these data have been available for a large sub-set of cases in England. We demonstrate that SNP typing can be used to detect and distinguish clusters representing likely sexual transmission in MSM from other transmission clusters in the community. Our approach can be used to deliver a more timely and appropriate public-health response. Our results are relevant to any country developing and implementing WGS for public-health surveillance and outbreak detection.

## Introduction

Shigellosis or severe bacillary dysentery is caused by four *Shigella* species (* Shigella flexneri *, *Shigella dysenteriae *, *Shigella sonnei* and *Shigella boydii *) transmitted via the faecal–oral route of infection [1, 2]. In England, shigellosis is often associated with travel to countries considered to be at ‘high-risk’ of enteric disease, primarily in South Asia or sub-Saharan Africa [1, 3]. Over the last 10 years, however, there has been evidence of increasing transmission through sex between men [4, 5]. This occurs through oral–anal sexual contact, and has been linked with specific sexual activities and drug-use behaviours among predominantly human immunodeficiency virus (HIV)-positive men who have sex with men (MSM) [6]. Our previous genomic studies have described MSM-associated *Shigella* species, and sub-lineages of those species, that show phenotypic and genotypic markers of high-level resistance to azithromycin (*mphA* and *ermB*), which is postulated to be linked to off-target effects from antibiotics prescribed for sexually transmitted infections [4, 7, 8].

The Gastrointestinal Bacteria Reference Unit (GBRU) at Public Health England (PHE) provides national microbiological reference services in England and Wales, including species identification and molecular typing for *Shigella* spp. Since August 2015, whole-genome sequencing (WGS) has been performed on all cultured isolates of *Shigella* spp. referred to the GBRU [9, 10]. SNP typing is used to aggregate isolates into single linkage clusters considered to represent cases that are linked through recent transmission events. This provides a tool used for public-health decision-making in near real-time to distinguish between linked cases that might be part of an evolving UK outbreak, thus requiring a robust infection-control response, and cases who likely acquired their infection whilst travelling abroad [11]. Limited routine demographic data are submitted to the GBRU alongside laboratory isolates (see Methods) and are used to classify clusters, but these data lack information on sexual identity and behaviour.

In the UK, *Shigella* spp. are notifiable infections [12]. In 2015, as part of routine case follow-up and investigation, PHE introduced a new questionnaire to standardize and expand the collection of exposure information on suspected cases of shigellosis. In response to our previous work, and for the first time, this included questions relating to sexual identity and recent sexual behaviour. We combined questionnaire data with WGS SNP typing data for *S. flexneri* cases to understand the sensitivity and specificity of WGS cluster categorization at a range of SNP thresholds for distinguishing clusters representing sexual transmission within MSM networks from other clusters representing community (non-sexual) transmission.

## Methods

This research was conducted through the National Institute for Health Research Health Protection Research Unit (NIHR HPRU) in Blood Borne and Sexually Transmitted Infections at University College London in partnership with PHE, in collaboration with the London School of Hygiene and Tropical Medicine, and the NIHR HPRU in Gastrointestinal Infections at the University of Liverpool in partnership with PHE, in collaboration with the University of East Anglia, the University of Oxford and the Quadram Institute.

### *S. flexneri* isolates

All *S. flexneri* isolates referred to the GBRU by hospital laboratories in England between August 2015 and July 2017 were included in this study. Demographic data submitted to the GBRU alongside laboratory isolates (name, date of birth, sex, postcode of residence and foreign travel history) were extracted from the GastroData Warehouse, an isolate-level database that houses GBRU laboratory results for confirmatory testing and typing of gastrointestinal bacteria. Duplicate isolates belonging to the same individual within a 2 week period were excluded.

### WGS and sequence analysis

Genomic DNA from bacterial isolates was extracted using the QiaSymphony DNA extraction platform (Qiagen). DNA was fragmented and tagged for multiplexing with Nextera XT DNA sample preparation kits (Illumina) and sequenced using the Illumina HiSeq 2500 platform at PHE. fastq reads from all sequences can be found in the PHE Pathogens BioProject PRJNA315192 at the National Center for Biotechnology Information Read Archive: https://www.ncbi.nlm.nih.gov/bioproject/315192.

Illumina reads were mapped to the reference strain [* S. flexneri* serotype 2a strain 2457T (GenBank accession no. AE014073.1)] using bwa-mem [13]. The resulting Sequence Alignment Maps (sam files) were sorted and indexed to produce Binary Alignment Maps (bam files) using SAMtools [14]. High-quality variant positions (mapping quality >30, depth >10, variant ratio >0.9) identified using gatk v2.6 in unified genotyper mode [15] were extracted and stored in SnapperDB [11]. Hierarchical single linkage clustering was performed on the pairwise SNP distance matrix at descending SNP thresholds (250, 100, 50, 25, 10, 5 and 0), as previously described [11]. The clustering is summarized as a ‘SNP address’ (a seven-digit code) that describes the cluster membership at each of the thresholds. For phylogenetic analyses, recombinant regions of the genome were identified using Gubbins v2.0 and masked [16]. Pseudosequences of polymorphic positions were used to create a maximum-likelihood tree using RAxML v8.2.8 under the General Time Reversible model using up to 1000 bootstrap replicates [17]. Tree annotation was performed using Interactive tree of life (iTOL) v4.3 [18, 19]. Antimicrobial-resistance determinants were detected using ‘GeneFinder’ [20] (https://github.com/phe-bioinformatics/gene_finder), a customized algorithm that utilizes Bowtie 2 [21] to map newly sequenced reads to a database of reference sequences, followed by SAMtools to create bam files [14]. Genes were defined as present if they represented 100 % of the reference sequence, with greater than 90 % nucleotide identity.

### Cluster classification

A cluster was defined as two or more cases where the isolates differed in their pairwise SNP comparisons by a defined distance. A threshold of 10-SNPs difference across the core-genome sequences of any two isolates is the current standard for defining likely transmission clusters in routine public-health surveillance of *Shigella* spp. at PHE. For this study, we performed the cluster analysis using a range of SNP thresholds (25, 10, 5 and 0) to test the sensitivity and specificity of these thresholds for identifying likely clusters that along with the submitted demographic data could be classified as either sexual or non-sexual transmission clusters.

Clusters were designated as ‘adult male’ (a pragmatic proxy for MSM transmission) if they comprised: (i) between two and five cases in total of which all were men aged 16 years or older, or (ii) more than five cases where at least 90 % were men aged 16 years or older. Clusters were designated as ‘household’ if two or more cases shared a living space (i.e. same postcode of residence, or had the same surname and were identified in the same Health Protection Team (HPT) area if the residential postcode was unavailable). HPTs are local PHE teams (21 in total) providing public-health advice and operational support for infectious disease outbreaks). Clusters were designated ‘travel-associated’ if they contained two or more cases and at least 50 % of these cases had reported travel to the same country (or world region) outside the UK. ‘Community’ clusters consisted of cases that did not meet any of the other classifications, including clusters of between two and five cases where at least one case was a woman, and clusters of more than five cases where the proportion of men aged 16 years or older was less than 90 %. Differences in cluster size and duration between ‘adult male’ clusters and other clusters were assessed using the Chi-square test for comparing two proportions.

### Standardized shigellosis exposure questionnaire

A standardized questionnaire was piloted from August 2015 to March 2017 by seven HPTs in England (three in London, four outside London) to follow-up cases of shigellosis (Fig. S1, available with the online version of this article). The questionnaire collected demographic information including sexual identity for individuals aged 18 years or older (heterosexual/straight, gay/lesbian, bisexual, other, don’t know/refuse to answer), sexual contact for adult men aged 18 years or older in the past 4 days [recent sexual contact (Yes/No) and if yes, was this with a man and/or a woman, or prefer not to answer], recent foreign travel (past 4 days), food and water consumption (past 4 days), clinical condition (date of onset, symptoms and hospitalization) and risk status (i.e. at increased risk of spreading the infection to others, such as individual being a food handler, healthcare worker or in contact with children aged 5 years or under [22].

### Validation of SNP clustering

Questionnaire data were linked to WGS records extracted from the GastroData Warehouse using name, date of birth, sex and full postcode of residence. Data were analysed for all clusters that contained at least one individual with a completed questionnaire. The questionnaire data were used as the gold standard to validate whether the designation of a cluster as ‘adult male’ (i.e. likely sexual transmission between MSM) was accurate. Overall sensitivity and specificity of the cluster tool was assessed for each threshold using the questionnaire data as the gold standard to classify cases as either MSM (gay or bisexual-identifying men, or men who reported recent same-sex sexual contact) or non-MSM (heterosexual men, women and children under the age of 18 years); men who did not report any information on sexual identity and recent sexual contact were excluded. We calculated the proportion of ‘adult male’ clustered cases that (i) self-identified as gay or bisexual men, (ii) reported recent same-sex sexual contact and (iii) reported recent foreign travel at a range of SNP thresholds.

### Antimicrobial resistance

The proportion of cases with *Shigella* spp. WGS data that showed genotypic markers of azithromycin resistance (*mphA* and *ermB*) was explored for all 10-SNP clusters that contained at least one individual with a completed questionnaire. The questionnaire data were used to calculate what proportion of these cases were self-identifying gay men.

## Results

### Description of WGS clusters

Between August 2015 and July 2017, there were 1006 referred *S *. *flexneri* isolates with WGS data in England, of which 563 aggregated into 92 single linkage clusters defined at the 10-SNP threshold cut-off. Most clusters were classified as ‘community’ (*n*=36) or ‘adult male’ (*n*=36), followed by ‘travel-associated’ (*n*=14) and ‘household’ (*n*=6). However, considering the overall number of cases across all clusters, then the ‘adult male’ clusters accounted for most of the case burden (68.9%, *n*=388), followed by ‘community’ (22.4%, *n*=126), ‘travel-associated’ (6.4%, *n*=36) and ‘household’ (2.3%, *n*=13). The median cluster size was 2 cases (range: 2 to 240 cases) and the median cluster duration (i.e. the time between the first and last reported cases) was 2 months (range: 1 day to 24 months).

‘Adult male’ clusters were generally larger; one-third (12/36) consisted of five or more cases, compared to 14.3 % (8/56) of other clusters (*P*=0.031). A total of 19.4 % (7/36) of ‘community’ clusters, 7.1 % (1/14) of ‘travel-associated’ clusters and none of the ‘household’ clusters consisted of five or more cases. Cluster duration was also greater for ‘adult male’ clusters compared to other clusters; half (18/36) of ‘adult male’ clusters persisted for 6 months or longer, compared to 25.0 % (14/56) of other clusters (*P*=0.014). A total of 36.1 % (13/36) of ‘community’ clusters, 7.1 % (1/14) of ‘travel-associated’ clusters and none of the ‘household’ clusters persisted for 6 months or longer. There was one dominant ‘adult male’ cluster that consisted of 240 cases occurring over a 24 month period; 61.9 % (240/388) of all ‘adult male’ cases were aggregated into this 10-SNP cluster.

### Description of questionnaire data

*S. flexneri* questionnaires were available for 190 cases, representing 37.8 % (190/503) of all cases reported to GBRU from the participating HPTs (42.4 % in London, 28.3 % outside London) and 21.9 % (190/868) of all cases reported nationally during the pilot period. A total of 75.3 % (*n*=143/190) of questionnaires were submitted by London HPTs. Self-reported sexual identity and recent sexual contact data were available for 88.9 % (*n*=152/171) and 92.5 % (*n*=123/133) of individuals with questionnaire data, respectively.

### Clusters with linked WGS and questionnaire data

Using a 10-SNP threshold, 34 single linkage clusters contained at least one individual with a completed questionnaire, representing 37.0 % (34/92) of all clusters that were detected nationally during the study period (Table 1). Of these clusters, 97.1 % (33) belonged to clonal complex (CC) 245, while 2.9 % (1) belonged to CC145. Combined they represented 22 ‘adult male’, 10 ‘community’ and 2 ‘travel-associated’ clusters. In total, these clusters contained 401 cases, representing 71.2 % (401/563) of all clustered cases nationally during the study period. Clusters classified as ‘adult male’ accounted for most of the cases (86.8 %, 348/401), followed by ‘community’ (10.7 %, 43/401) and ‘travel-associated’ (2.5 %, 10/401) clusters. At the 10-SNP threshold, 26.4 % (106/401) of clustered cases had their isolates linked to questionnaire data. The size and distribution of clusters varied depending on the SNP threshold. Table 1 presents a summary of clustered cases reported for each SNP threshold cut-off, and the proportion linked to a questionnaire.

**Table 1.** Size and distribution of clusters by SNP threshold cut-off and cluster classification

	25-SNP threshold (27 clusters, 515 cases)			10-SNP threshold (34 clusters, 401 cases)			5-SNP threshold (39 clusters, 286 cases)				0-SNP threshold (25 clusters, 69 cases)		
	Adult male	Community	Travel-associated	Adult male	Community	Travel-associated	Adult male	Community	Travel-associated	Household	Adult male	Community	Travel-associated
No. of clusters	10	14	3	22	10	2	28	8	2	1	20	3	1
No. of cases	424	76	15	348	43	10	246	28	10	2	51	11	7
Cluster size													
Median (range)	4 (2-313)	3 (2-22)	5 (2-8)	4 (2-240)	3 (2-8)	5 (2-8)	3 (2-144)	3 (2-8)	5 (2-8)	–	2 (2-5)	2 (2-7)	–
2	4	5	1	9	5	1	11	4	1	1	13	2	0
3-4	2	4	0	3	1	0	9	2	0	0	6	0	0
5-9	0	3	2	6	4	1	5	2	1	0	1	1	1
10-19	2	1	0	3	0	0	2	0	0	0	0	0	0
20+	2	1	0	1	0	0	1	0	0	0	0	0	0
No. (%) of cases with a questionnaire	106 (25.0)	18 (23.7)	3 (20.0)	89 (25.6)	15 (34.9)	2 (20.0)	74 (30.1)	11 (39.3)	2 (20.0)	2 (100.0)	25 (49.0)	3 (27.3)	1 (14.3)

### SNP clustering validation

**Fig. 1.**
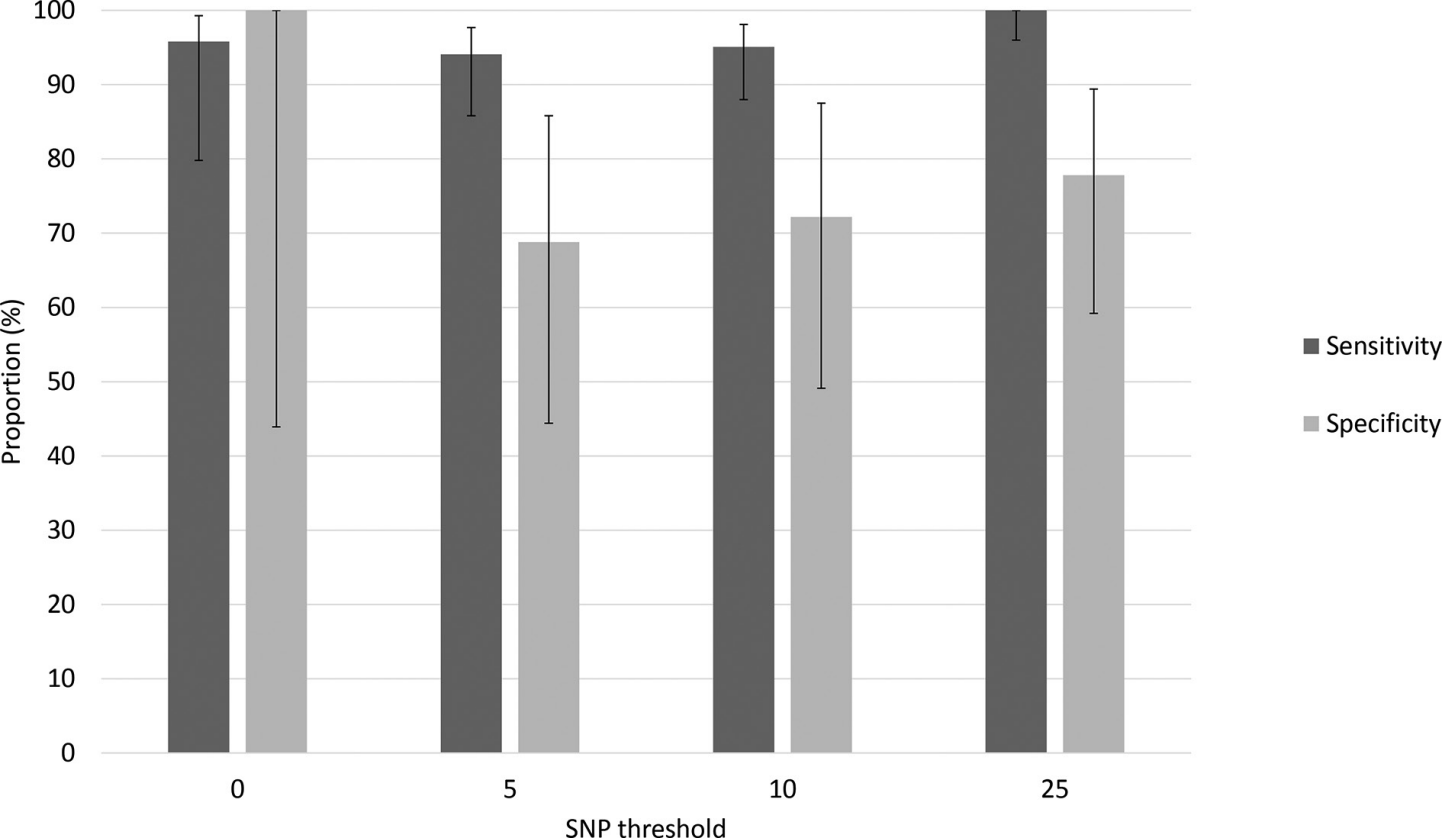
Sensitivity and specificity of the cluster classification tool at different SNP thresholds using the questionnaire data as the gold standard. Sensitivity represents the proportion of MSM that belonged to an ‘adult male’ cluster and specificity represents the proportion of non-MSM (heterosexual men, women and children under the age of 18 years) that belonged to a ‘community’, ‘travel-associated’ or ‘household’ cluster. Error bars represent 95 % confidence intervals.

Table 2 presents the proportion of clustered cases for each SNP threshold cut-off by (i) sexual identity, (ii) recent sexual contact and (iii) recent foreign travel. For clusters at all SNP thresholds, there were adult men who did not provide information on sexual identity (for example, *n*=9 at the 10-SNP threshold) and/or recent sexual contact (*n*=7 at the 10-SNP threshold) that clustered with men identifying as gay. Overall sensitivity and specificity of the cluster classifications at a range of SNP thresholds are presented in Fig. 1. At the 10-SNP threshold, 95.1 % (77/81) of MSM (gay-identifying men or men who reported recent same-sex sexual contact) belonged to an ‘adult male’ cluster and 4.9 % (4/81) to a ‘community’ cluster; most (74.1 %; 60/81) reported recent same-sex sexual contact. Among the non-MSM cohort, 27.8 % (5/18, all heterosexual men) belonged to an ‘adult male’ cluster, 61.1 % (11/18) to a ‘community’ cluster and 11.1 % (2/18) to a ‘travel-associated’ cluster. At the 25-SNP threshold, all MSM belonged to an ‘adult male’ cluster and for non-MSM, 22.2 % (6/27, all heterosexual men) belonged to an ‘adult male’ cluster, 66.7 % (18/27) to a ‘community’ cluster and 11.1 % (3/27) to a ‘travel-associated’ cluster.

**Table 2.** Sexual identity, recent sexual contact and foreign travel for clustered cases with a completed questionnaire

	25-SNP threshold (*n*=127)			10-SNP threshold (*n*=106)			5-SNP threshold (*n*=89)				0-SNP threshold (*n*=29)		
	Adult male *n*=106	Community *n*=18	Travel *n*=3	Adult male *n*=89	Community *n*=15	Travel *n*=2	Adult male *n*=74	Community *n*=11	Travel *n*=2	Household *n*=2	Adult male *n*=25	Community *n*=3	Travel *n*=1
**Sexual identity**													
Gay men	90 (84.9)	0	0	75 (84.3)	4 (26.7)	0	63 (85.1)	4 (36.4)	0	0	23 (92.0)	1 (33.3)	0
Heterosexual men	6 (5.7)	3 (16.7)	2 (66.6)	5 (5.6)	1 (6.7)	1 (50.0)	5 (6.76)	0	1 (50.0)	1 (50.0)	0	0	1 (100.0)
Adult men*	10 (9.4)	0	0	9 (10.1)	0	0	6 (8.1)	1 (9.1)	0	0	2 (8.0)	0	0
Lesbian	0	0	0	0	0	0	0	0	0	0	0	0	0
Heterosexual women	0	11 (61.1)	0	0	9 (60.0)	1 (50.0)	0	6 (54.5)	0	1 (50.0)	0	2 (66.7)	0
Adult women*	0	2 (11.1)	1 (33.3)	0	0	0	0	0	1 (50.0)	0	0	0	0
Other (<18 years old)	0	2 (11.1)	0	0	1 (6.7)	0	0	0	0	0	0	0	0
**Recent sexual contact†**													
With man	65 (61.3)‡	0	0	56 (62.9)‡	4 (80.0)	0	45 (60.8)§	4 (80.0)	0	0	21 (84.0)	1 (100.0)	0
With woman	3 (2.8)	2 (66.7)	0	2 (2.2)	1 (20.0)	0	2 (2.7)	0	0	1 (100.0)	0	0	0
Gender not disclosed	2 (1.9)	0	0	2 (2.2)	0	0	1 (1.4)	1 (20.0)	0	0	0	0	0
No sexual contact	30 (28.3)	1 (33.3)	2 (100.0)	24 (27.0)	0	1 (100.0)	21 (28.4)	0	1 (100.0)	0	3 (12.0)	0	1 (100.0)
Not known	6 (5.7)	0	0	5 (5.6)	0	0	5 (6.8)	0	0	0	1 (4.0)	0	0
**Foreign travel||**													
Europe	11 (10.4)	0	0	9 (10.1)	1 (6.7)	0	7 (9.5)	1 (9.1)	0	0	2 (8.0)	1 (33.3)	0
Caribbean	1 (0.9)	0	0	1 (1.1)	0	0	1 (1.4)	0	0	0	0	0	0
Middle East	2 (1.9)	1 (5.6)	1 (33.3)	2 (2.2)	0	1 (50.0)	1 (1.4)	0	1 (50.0)	0	0	0	1 (100.0)
North Africa	0	2 (11.1)	0	0	1 (6.7)	0	0	1 (9.1)	0	0	0	0	0
South Asia	0	4 (22.2)	1 (33.3)	0	1 (6.7)	1 (50.0)	0	0	1 (50.0)	0	0	0	0
Sub-Saharan Africa	1 (0.9)	4 (22.2)	0	1 (1.1)	3 (20.0)	0	1 (1.4)	3 (27.3)	0	0	0	1 (33.3)	0
No or unknown recent travel	91 (85.9)	7 (38.9)	1 (33.3)	76 (85.4)	9 (60.0)	0	64 (86.5)	6 (54.5)	0	2 (100.0)	23 (92.0)	1 (33.3)	0

*Sexual identity not reported.

†Denominator includes adult men (≥18 years) only.

‡Includes 2 men who did not disclose their sexual identity (i.e. their sexual identity is reported here as ‘adult men’) but who reported recent same-sex sexual contact.

§Includes 1 man who did not disclose his sexual identity (i.e. his sexual identity is reported here as ‘adult men’) but who reported recent same-sex sexual contact.

||Foreign travel as recorded on the questionnaire; data missing for 1 case.

**Fig. 2.**
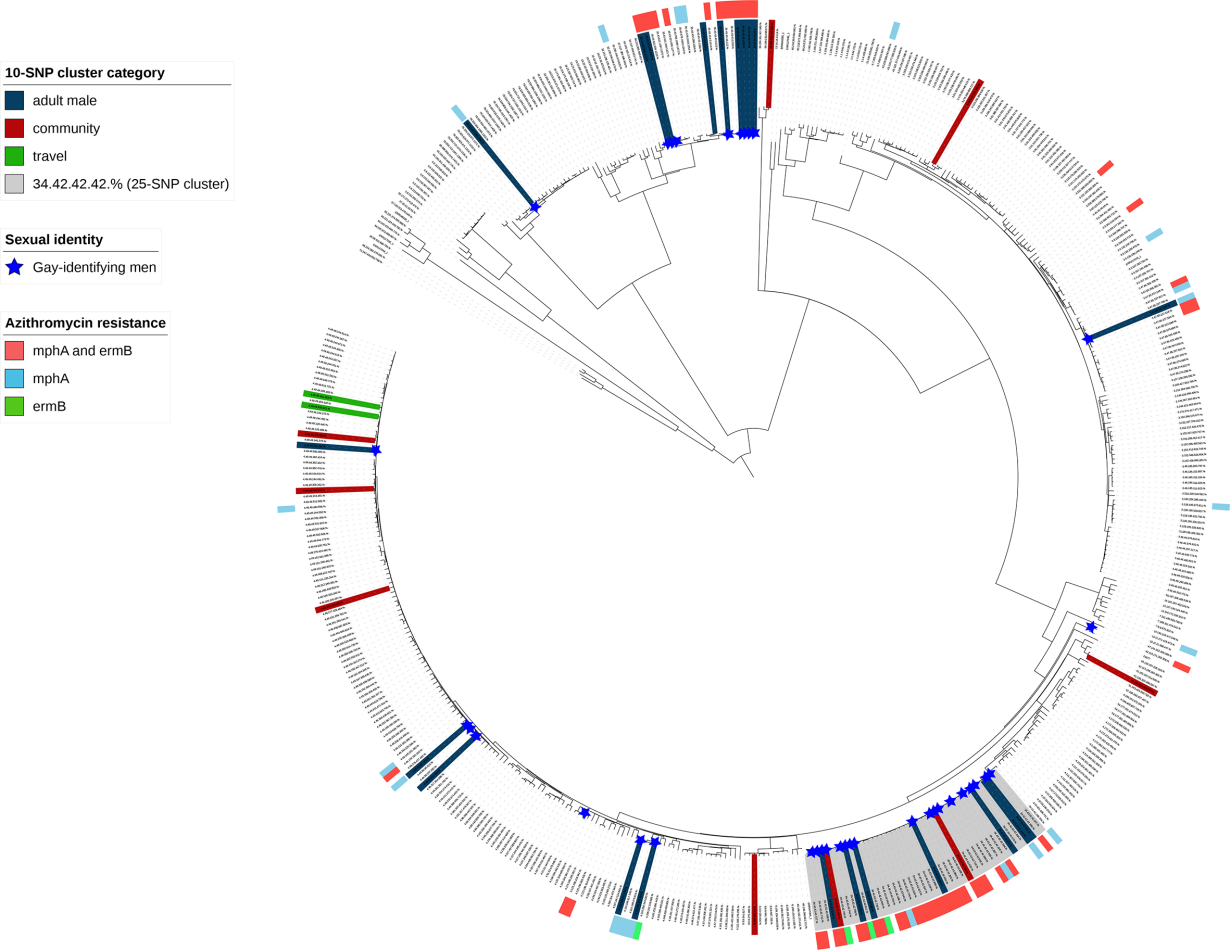
Phylogeny of *S. flexneri* CC245 showing the distribution of clusters, genotypic markers of azithromycin resistance and gay-identifying men. Mid-point rooted maximum-likelihood phylogenetic tree showing a single representative isolate from each 10-SNP single linkage cluster (*n*=474) for CC245 during the study period (*n*=926) and seven reference strains for each phylogenetic group [36]. The number of isolates represented by each tip ranges from 1 to 240. Single linkage clusters (i.e. 2 or more cases) containing at least one case with a questionnaire are coloured at the tips according to cluster classification (33 clusters representing 394 cases). Genotypic markers of azithromycin resistance are shown as a coloured track on the outside of the tree. Isolates from gay-identifying men are shown as blue stars. An ‘adult male’ 25-SNP cluster is highlighted in grey.

For some clusters, the classification changed when performing the validation analysis at different SNP thresholds providing a deeper understanding of likely transmission routes. Three ‘community’ clusters (two 10-SNP, one 5-SNP) were part of a larger 25-SNP ‘adult male’ cluster. These clusters did not meet the criteria to be defined as ‘adult male’; for clusters comprising between two and five cases at least one case was female, and for clusters comprising more than 5 cases, less than 90 % were men aged 16 years or older (Table 3). All isolates in this 25-SNP ‘adult male’ cluster (highlighted in grey in Fig. 2) belonged to the same phylogenetic clade, for which 10-SNP clustering identified multiple discrete clusters (7 ‘adult male’; 2 ‘community’) (Fig. S2). Overall, 74.2 % (69/93) of isolates (clustered and non-clustered) from gay-identifying men belonged to this clade (Fig. 2).

**Table 3.** Changes in cluster classification at a range of SNP threshold cut-offs

ID	SNP threshold	SNP address	Classification	Total cases	M:F ratio*	No. (%) of adult men	No. (%) of gay-identifying men†	No. (%) of foreign travel cases‡	Min. SNP	Max. SNP	Median SNP
**1**	25	34.42.42.42.%	Adult male	313	301 : 8	301 (97.4)	69/81 (85.2)	20 (6.4)	0	47	21
	10	34.42.42.42.344.%	Community	7	6 : 1	6 (85.7)	3/3 (100)	0	1	11	5
	5	34.42.42.42.344.396.%	Community	5	4 : 1	4 (80.0)	3/3 (100)	0	1	7	4
**2**	25	34.42.42.42.%	Adult male	313	301 : 8	301 (97.4)	69/81 (85.2)	20 (6.4)	0	47	21
	10, 5, 0	34.42.42.42.537.%	Community	2	1 : 1	1 (50.0)	1/1 (100)	0	0	0	0
**3**	25	34.42.42.42.%	Adult male	313	301 : 8	301 (97.4)	69/81 (85.2)	20 (6.4)	0	47	21
	10	34.42.42.42.42.%	Adult male	240	232 : 5	232 (97.9)	48/56 (85.7)	15 (6.3)	0	38	17
	5	34.42.42.42.42.526.%	Community	4	3 : 1	3 (75.0)	0/1§	0	2	7	4
**4**	25	4.49.49.129.%	Community	10	4 : 5	4 (44.4)	0/2	3 (30.0)	3	20	13
	10	4.49.49.129.325.%	Community	8	2 : 5	2 (28.6)	0/2	2 (25.0)	3	17	11
	5	4.49.49.129.324.663.%	Household	2	1 : 1	1 (50.0)	0/2	0	3	3	3

*Ratio of males to females.

†Denominator includes individuals with a questionnaire only.

‡Foreign travel as reported through questionnaires or laboratory records (for those without a questionnaire).

§Adult man who did not report sexual identity and preferred not to disclose gender of partner.

### Genotypic markers of azithromycin resistance

Genotypic markers of azithromycin resistance were detected in the genomes linked to 84.5 % (294/348; 29 *mphA*, 11 *ermB,* and 254 *mphA* and *ermB*) of cases belonging to ‘adult male’ clusters and 20.9 % (9/43; all *mphA* and *ermB*) of cases belonging to ‘community’ clusters at the 10-SNP threshold (Fig. 2). Of those cases where a questionnaire was completed, 83.3 % (65/78) and 100 % (4/4) within ‘adult male’ and ‘community’ clusters, respectively, were gay-identifying men. The other 16.7 % (*n*=13) comprised five heterosexual-identifying men, two of whom reported recent sexual contact with a woman, and eight adult men who did not provide information on sexual identity, although two of whom reported recent sexual contact with a man. The four gay-identifying men within the ‘community’ clusters belonged to the two ‘community’ clusters that were part of a larger 25-SNP ‘adult male’ cluster (see Table 3 for details of these clusters).

## Discussion

This is the first time that robust data on sexual identity and behaviour have been available for a large sub-set of *Shigella* cases in England, and we have been able to utilize this data to validate a real-time SNP clustering algorithm. We provide evidence that SNP clustering using limited routine demographic data can distinguish clusters representing likely sexual transmission in MSM from other clusters representing community (non-sexual) transmission, thereby informing rapid and appropriate public-health responses.

The main limitation of this study is that behavioural data were only available for HPTs participating in a national pilot study. In our study, a higher proportion of clusters were ‘adult male' compared to the national cluster data, and a higher proportion of all clustered cases belonged to these ‘adult male’ clusters. This bias towards ‘adult male’ cases may reflect the larger MSM populations within the participating HPTs, including London, Manchester and Brighton and Hove [23]. It is likely that a higher proportion of *Shigella* cases in these regions were part of ‘adult male’ clusters. As a result, our analysis may have overestimated the proportion of cases transmitted through sexual contact in MSM compared to other transmission routes. This could have biased our cluster validation analysis if the HPTs were more likely to follow-up MSM cases, or if the HPTs not included in the pilot were more likely to see ‘adult male’ clusters that were linked through community (non-sexual) transmission. It is also important to note that sexual identity and recent sexual contact were only collected for individuals aged 18 years or older, whereas the ‘adult male’ cluster classification used a cut-off of 16 years or older. The impact of this on our validation analysis is negligible due to the small number of individuals falling within this 2 year age gap.

Our study is restricted to cases who presented to healthcare, with a stool sample collected for investigation by local hospital laboratories and referral to the GBRU for molecular typing; approximately two-thirds of *Shigella* spp. isolated at local hospital laboratories are referred to the GBRU [24]. This under-ascertainment might have led to bias in our study, because a range of factors are likely to influence whether an isolate was included, such as patterns of health-seeking behaviour, testing practices and gender disparities in travel, food consumption or childcare [25]. A large population-based cohort study of infectious intestinal disease in England found that presenting to healthcare was associated with severity of illness, recent foreign travel, educational level and lower socioeconomic status [26]. Furthermore, an analysis of *Shigella* spp. surveillance data from the USA found that the odds of severe shigellosis were higher for men compared to women among adults aged 18–49 years old, and for men compared to women among individuals diagnosed with *S. flexneri *. However, the role of sexual behaviour was not assessed [27]. Collectively, such factors are more likely to lead to an overestimation in the proportion of cases transmitted through sexual contact.

In our study, we confirmed that most cases belonged to ‘adult male’ clusters that predominantly consisted of MSM with no or unknown recent travel history. One dominant ‘adult male’ cluster persisted for 24 months, consistent with ongoing sexual exposure and potential for reinfection within large and dense sexual networks [6, 28–30]. Interestingly, up to 28 % of gay men within the ‘adult male’ clusters did not report recent sexual contact, which could suggest transmission through community (non-sexual) contact, a delay in symptom onset (due to an incubation period of longer than 4 days), relapse of a previously acquired chronic infection, or misreporting of recent behaviour. Of concern, and in contrast to other clusters, genotypic markers of azithromycin resistance were detected in most cases belonging to ‘adult male’ clusters. High-level azithromycin resistance in MSM transmission networks has been shown to play a role in driving the spread of *Shigella* infection [8], and this raises concerns about future treatment options for *Shigella* and the wider global problem of AMR.

Our findings may inform best practice for *Shigella* cluster investigations in any country developing and implementing WGS for surveillance and/or outbreak detection. However, this methodology is dependent on building a comprehensive library of contextual WGS data against which to classify new cases. Our analysis showed that real-time WGS of *Shigella* can be used to detect likely clusters of sexual transmission in MSM, but also showed that a flexible approach to SNP thresholds, or one that encompasses phylogenetic context, is important to fully understand the underlying transmission network and likely transmission route, particularly in clusters comprising fewer than 10 cases where at least one is female. In addition, persistent transmission networks sustained through sexual contact over longer periods of time will naturally exhibit greater genetic diversity, which should be considered when investigating clusters. In practice, restricting the SNP threshold to 10-SNPs may lead to subsequent investigation of clusters that appear to be part of different sexual networks, or the delivery of non-targeted public-health messages. In addition, community (non-sexual) transmission between MSM and non-MSM population groups, although currently limited to small numbers, should not be ignored.

GBRU typing data are used for routine surveillance and are currently assessed on a weekly basis for active clusters. The decision to further investigate a cluster depends on several factors, including cluster classification, duration, size and geographical spread. Cluster-specific interventions are not usually required for ‘adult male’ clusters that are geographically and temporally dispersed. In collaboration with partners including the British Association for Sexual Health and HIV (BASHH), the Terrence Higgins Trust (THT) and the LGBT Foundation, PHE provides public-health messages targeted towards MSM and clinicians that aim to raise awareness of *Shigella* and offer advice on what men can do to lower their risk (e.g. washing hands and genitals before and after sex, using condoms for risk practices and avoiding the use of shared sex toys) [31]. Despite this, it is not clear whether these public-health messages are effective. In 2016, a London-wide sexual-health promotion campaign focussed on raising awareness through social media, and through posters and leaflets displayed in sexual-health clinics. The campaign evaluation found that overall awareness among MSM remained low (29%) [32]. This study provides an additional route to provide appropriate and targeted healthcare advice to those who may not identify as gay or bisexual, or reveal same-sex sexual contact, but who are likely to be part of an MSM network and, therefore, at higher risk of acquiring shigellosis, as well as other sexually transmitted infections and HIV infection. Identification of sexual transmission is important to ensure prompt and onward referral to sexual-health clinics.

The utility of SNP clustering for improving case ascertainment during outbreak investigations, and revealing linked cases not identified prior to the implementation of WGS, has been described previously [33–35]. However, detection and classification of WGS clusters should complement traditional epidemiological methods, including questionnaire completion, which can help to unravel the characteristics of the cluster and describe how transmission might be occurring. For example, epidemiological data may identify individuals who share a common source, such as attendance at a specific sex-on-premises venue, and these clusters may be amenable to public-health action including improvements in hygiene and the provision of condoms. The addition of epidemiological data to SNP clustering data may also reveal novel links between MSM and other populations in the community, particularly where MSM are within a known risk group (e.g. food handlers or healthcare workers). Sustained transmission in sexual networks could seed community acquired outbreaks, which is a concern given the high rates of antimicrobial resistance in the MSM population. Therefore, these clusters may be prioritized for public-health action.

## Data bibliography

Public Health England. National Center for Biotechnology Information Read Archive, BioProject number PRJNA315192 (2018).

## Supplementary Data

Supplementary File 1

Supplementary File 2
