# Supplementary material for: Use of whole-genome sequencing to identify clusters of Shigella flexneri associated with sexual transmission in men who have sex with men in England: a validation study using linked behavioural data
Source: Microb Genom. 2019 Nov 4;5(11):e000311. doi: 10.1099/mgen.0.000311 (PMC6927305; doi:10.1099/mgen.0.000311)
Supplement: Supplementary File 2 [file mgen-5-311-s002.pdf]

Supplementary Material

Supplementary File 1. Standardised shigellosis exposure questionnaire

Standardised shigellosis exposure questionnaire used by Health Protection Teams (HPTs) participating in a national pilot

Shigella *flexneri/dysenteriae/boydii* questionnaire

1: QUESTIONNAIRE DETAILS

Interviewer name:		Interview date :	
Interviewer organisation:		Interviewer Telephone:	
Person interviewed name and relationship to case:			

2: CASE CLASSIFICATION AND ID

PHE reference no. e.g. HPZone					
Environmental health ID no.(if relevant)					
If relevant: GUM clinic name		GUM clinician		GUM clinic no.	
Local laboratory result: Culture					
PCR					
Local laboratory specimen number		Local Laboratory name			
Reference laboratory result					

		Confirmed	Probable	Possible
Classification	*Shigella flexneri*	☐	☐	☐
	Shigella dysenteriae	☐	☐	☐
	Shigella boydii	☐	☐	☐
	Shigella species	☐	☐	☐

3: PERSONAL AND IDENTIFYING DETAILS

First name:		Family name/Surname:			
Address:					
Postcode:		Tel (h):		Tel (m):	
Email:					
Sex:	M ☐ F ☐	Date of birth (dd/mm/yyyy):		Age:	yrs
NHS No:		GP name:			
GP address:				GP Tel:	
Are there any children living in the household? (other than the case)		Y ☐	N ☐	How many? ☐	

Public Health England is committed to ensuring all individuals are treated equally and fairly, and therefore we ask all people the following questions about their ethnicity and sexual orientation. This also helps us to identify and investigate outbreaks.

**Ethnicity:**				
White	☐ British	☐ Irish		☐ Other (please state)
Mixed	☐ White/Black Caribbean	☐ White/Black African	☐ White/Asian	☐ Other (please state)
Asian/Asian British	☐ Indian	☐ Pakistani	☐ Bangladeshi	☐ Other (please state)
Black/Black British	☐ Caribbean	☐ African		☐ Other (please state)
Chinese	☐ Chinese			
Other	☐ (please state)			

**Sexual identity (if over 18 years old)**
I will now read out a list of terms people sometimes use to describe how they think of themselves.
☐ Heterosexual or straight
☐ Gay or lesbian
☐ Bisexual
☐ Other
As I read the list again please say 'yes' when you hear the option that best describes how you think of yourself.(Pause briefly after each option during second reading).
☐ Spontaneous don't know/refusal

4: RISK GROUPS

Occupation			
Work/School/Nursery name:		Tel:	
Contact person			
Address:			
Postcode:		Date last attended:	

Does the patient fit into any of the following categories (tick all that apply)?:		
**Group A**	☐	Any person of doubtful personal hygiene or with unsatisfactory toilet, hand washing or hand drying facilities at home, work or school. Particular consideration should be given as to whether individual infant-school-aged children (aged 6 or 7 years) are able to satisfactorily observe good personal hygiene. Health protection personnel (LA and HPU) should agree locally on how to make this assessment in engagement with parents or teachers/carers.
**Group B**	☐	All children aged five years old or under including those who attend school, pre-school, nursery or other childcare or minding groups.
**Group C**	☐	People whose work involves preparing or serving unwrapped food to be served raw or not subjected to further heating.
**Group D**	☐	Clinical, social care or nursery staff who work with young children, the elderly, or other particularly vulnerable people, and whose activities increase the risk of transferring infection via the faecal-oral route. Such activities include helping with feeding or handling objects that could be transferred to the mouth
**No risk group**	☐	

5: CLINICAL DETAILS

Onset date:		Still ill:		If no, →	Duration of illness (days):	.	
Symptoms:	Diarrhoea Yes ☐ No ☐			Vomiting Yes ☐ No ☐			
	Fever Yes ☐ No ☐			Blood in stools Yes ☐ No ☐			
	Abdominal pain Yes ☐ No ☐			Mucus in stool Yes ☐ No ☐			
Healthcare sought from:	☐ GP visit	☐ A&E	☐ Sexual health clinic	☐ Other (specify):			
Date of stool sample:							
Admitted to hospital for this illness:	Yes ☐ No ☐			Admission date:			
Hospital name:				Duration of stay (d):			
Treated with antibiotics:	Yes ☐ No ☐			If Y, specify:			
Has the patient heard of *Shigella* before?	Yes ☐ No ☐						

6: TRAVEL

6.1 In the **FOUR** *(boydii, flexneri)* or **SEVEN** (*dysenteriae)* days prior to illness, did you arrive or return to the UK from **ABROAD**?	Yes ☐ No ☐
Specify countries visited (from most recent)	

**Country/Region**	**Date arrived**	**Date departed**	**Details**

6.1 In the **FOUR** *(boydii, flexneri)* or **SEVEN** (*dysenteriae)* days prior to illness, did you travel elsewhere **WITHIN** the UK?

**Town/Resort**	**Date arrived**	**Date departed**	**Details**

7: OTHER RISK FACTORS

7.1 In the **FOUR** *(boydii, flexneri)* or **SEVEN** (*dysenteriae)* days prior to illness, did you have any contact with:

Anyone with diarrhoea?	Yes ☐ No ☐	If yes, details:	
Children under 5 years?	Yes ☐ No ☐	If yes, details:	
Visitors from UK or overseas?	Yes ☐ No ☐	If yes, details:	
Attend a gym/ swimming pool/other communal sports facilities?	Yes ☐ No ☐	If yes, where?	

8: SEXUAL CONTACT (ONLY for MALE cases aged18 years or older. If under 18 years please go straight to section 9). Please ask about sexual contact regardless of travel history or other identified risk factors.

8.1 We ask all adult men with Shigella about sexual contact as Shigella can be sexually transmitted via the faecal-oral route. Men who have sex with men (MSM) are at greater risk of acquiring the infection than others and there have been outbreaks of shigella among the MSM population.

Did you have sexual contact with anyone in the **FOUR** (*boydii, flexneri)* or **SEVEN** (*dysenteriae)* days prior to illness			Yes ☐ No ☐
**IF** yes, was this with a	Male ☐ and/or	Female ☐	Prefer not to answer ☐

8.2 Public Health Advice: If the case is a MSM - please remember to provide them information on how to reduce transmission as per PHE leaflet (link below) and recommend they attend their local sexual health clinic for STI screening.

https://www.gov.uk/government/uploads/system/uploads/attachment_data/file/323532/Shigella_leaflet.pdf

If the case has been abroad to areas with a high risk for shigella infection in the FOUR *(boydii, flexneri)* or SEVEN (*dysenteriae)* days prior to illness, AND/OR sexual transmission has been identified as a likely source, please go straight to section 10.

High risk area - South America, Asia (including the Middle East) and Africa: Low risk areas - Europe, North America and Australia:

9: FOOD AND WATER HISTORY

9.1 Food prepared at home

Please list all food eaten in the **FOUR** *(boydii, flexneri)* or **SEVEN** (*dysenteriae)* before you became unwell. Describe what was eaten and when in the appropriate section on the chart. If possible give details of the shop where it was bought. **Routine cooking kills *Shigella* bacteria so particular attention should be given to raw and uncooked food, as well as baby foods.**

Days pre-onset	Date	Breakfast	Lunch	Evening meal	Snacks
1					
2					
3					
4					
5					
6					
7					

9.2 In the **FOUR** *(boydii, flexneri)* or **SEVEN** (*dysenteriae)* days before you became unwell, did you eat any food that was not prepared at home, either in this country or abroad (e.g. hotels, restaurants, cafes, pubs; school and work canteens; takeaways, fast food outlets; barbecues and picnics; social events; other people's homes)? If yes, enter details below

Date	Description of food	Establishment where food obtained

9.3 In the **FOUR** *(boydii, flexneri)* or **SEVEN** (*dysenteriae)* days before you became unwell, what was the source of your drinking water? (tick all that apply)

Mains ☐	Private ☐	Bottled ☐
Filtered ☐	Well ☐	Spring ☐
Other (specify) ☐		

10: PLEASE COMPLETE CONTACT SHEET ON THE FOLLOWING PAGE

11: FURTHER INFORMATION

Is this case part of an outbreak?	Yes ☐ No ☐	**Setting**	
Are there any other possible exposures to infection not already discussed			
May we contact you again if we need to ask any further questions?		Yes ☐	No ☐

Please remember for provide public health advice to reduce risk of ongoing transmission

CONTACTS

PHE (HPZ) REF NUMBER: CASE NAME:

***Household/significant contacts: List everyone who lives in the same accommodation or shares the same kitchen, bathroom or toilet**.
^Type of symptom: D= Diarrhoea; F= Fever; Ab= Abdo pain; V= Vomiting; M= mucus in stool; and BS= Bloody stool.

	Name	Address	Date of birth	Relatio-nship to case*	Gen-der (M/F)	Sympt oms (Y/N)	Type of Symptom	Date of Onset	Risk Group (A/B/C/D /No)	GP Details	Exclusio n req'd (Y/N)	Contact advised re. excl-usion (Y/N)
1												
2												
3												
4												
5												

	Name	Address	Date of birth	Relatio-nship to case*	Gen-der (M/F)	Sympt oms (Y/N)	Type of Symptom	Date of Onset	Risk Group (A/B/C/D /No)	GP Details	Exclusio n req'd (Y/N)	Contact advised re. excl-usion (Y/N)
6												
7												
8												

Significant contact is defined as household/workplace/school contact and/or those who have been exposed to the similar circumstances as the case i.e. travelled with the case.

Supplementary File 2. Phylogeny of 25-SNP ‘adult male’ cluster 34.42.42.42.%

Mid-point rooted maximum likelihood phylogenetic tree containing all isolates belonging to an ‘adult male’ 25-SNP cluster (34.42.42.42.%, N=313). Isolates are labelled by SNP address. 10-SNP single linkage clusters containing at least one case with a questionnaire are coloured at the tips by cluster (9 clusters, 273 cases in total). Branch tips that are not coloured represent cases that did not cluster with another case at the 10-SNP threshold (n=33 cases), or 10-SNP single linkage clusters that did not contain a case with a questionnaire (2 ‘adult male’ clusters, 7 cases in total). Selected demographic data and sexual risk are represented as coloured strips. Sexual risk data are presented for cases with a questionnaire.

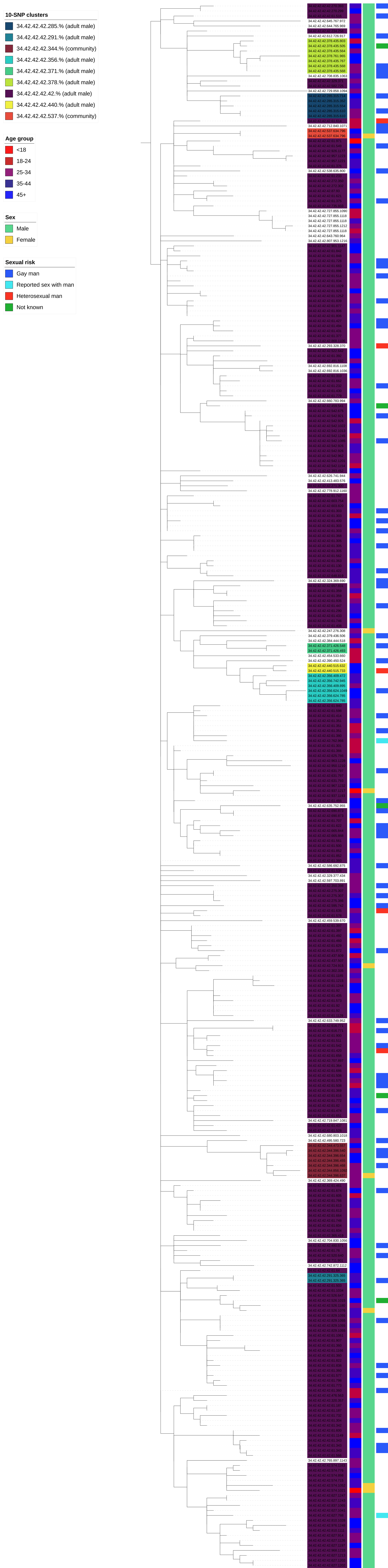
10-SNP clusters
34.42.42.42.285.% (adult male)
34.42.42.42.291.% (adult male)
34.42.42.42.344.% (community)
34.42.42.42.356.% (adult male)
34.42.42.42.371.% (adult male)
34.42.42.42.378.% (adult male)
34.42.42.42.42.% (adult male)
34.42.42.42.440.% (adult male)
34.42.42.42.537.% (community)
Age group
<18
18-24
25-34
35-44
45+
Sex
Male
Female
Sexual risk
Gay man
Reported sex with man
Heterosexual man
Not known
